# Beyond Digital Interventions: Human-Centered Design for Adolescents’ Type 1 Diabetes Management in LMICs

**DOI:** 10.1145/3700794.3700808

**Published:** 2025-03-11

**Authors:** Susan Wyche, Jennifer Olson, Mary Karanu, Lilian Mathu, Eric Omondi

**Affiliations:** Media & Information, Michigan State University, USA; Media & Information, Michigan State University, USA; Rural Outreach Africa, Kenya; Independent Researcher, Kenya; Diabetes Management & Information Centre, Kenya

**Keywords:** Design, Diabetes, Kenya, mHealth, mobile phones, user studies

## Abstract

Human-centered design (HCD) centers users’ perspectives in the technology design process. This approach is widely used in ICTD to develop digital interventions for people in low- and middle-income countries (LMICs), including mobile health (mHealth) applications. However, primarily using HCD to develop digital interventions limits understanding of how to design interventions for users who do not regularly use digital technologies, particularly smartphones. In this article, we contribute a case study documenting our collaboration with Kenyan adolescents and their caregivers to develop a prototype paper-based diary to support type 1 diabetes (T1D) management. We describe how outcomes from the project’s user research and ideation phases—in particular, findings from two design workshops—contributed to the development of the diary. Our findings motivate a discussion about considering alternative HCD outcomes: in particular, non-digital interventions, new knowledge creation, and community-building

## 1 INTRODUCTION

Human-centered design (HCD) is an established approach to technology design that prioritizes users’ needs in the design process. This creative design strategy is widely used in Information Communication Technology and Development (ICTD) [24, 44]. Enthusiasm for HCD has broadened beyond ICTD, and the approach underlies the development of global health interventions—in particular, mobile health, or “mHealth” applications, which are regarded as promising for improving health outcomes in low- and middle-income countries (LMICs) [21, 45]. However, using HCD primarily to develop digital interventions limits our understanding of how to design interventions for users who do not own or regularly use smartphones [6, 15]. In this paper, we describe our implementation of HCD to develop a personal paper-based diary that supports blood sugar logging and other activities related to type 1 diabetes (T1D) management by adolescents. We conducted this research in an LMIC—specifically, Kenya in East Africa, where the incidence of T1D is rising [2]. This chronic condition, which is frequently diagnosed in adolescents requires careful management.

The research presented here is part of a longer-term project. We describe our implementation of HCD—from the understanding or formative research phase to the ideation phase, and the development of an early prototype. We primarily used these HCD methods in our study: interviews, photo study, and design workshops. 24 youths with T1D and their caregivers participated in this project. We conducted research at sites in urban Nairobi and rural Vihiga County.

Our contributions to ICTD include a case study documenting our HCD process, a novel prototype of a personal paper-based diary with an educational poster insert, and a discussion about considering alternative HCD outcomes in ICTD. In particular, we draw attention to these outcomes: non-digital solutions, new knowledge creation, and community-building. Case studies like this one are necessary to facilitate the replication of HCD projects and to inform critical discussions about using the approach in LMICs.

## 2 BACKGROUND AND RELATED WORK

### 2.1 Overview: Human-Centered Design (HCD)

“Human-centered design” is used interchangeably with “user-experience design” and “design thinking”, due to these terms’ shared emphasis on centering the people you are designing for [21, 24]. The approach originated from research conducted in the 1970s in ergonomics, computer science, and artificial intelligence [3]. It has evolved from an engineering-based approach that focused on improving “man-machine fit”, to one that broadly encompasses placing “our understanding of people (. . .) at the forefront in the design of new technology” [2, 16].

HCD is a flexible but disciplined multi-phased approach that generally begins with the understanding phase; that is, designers learn about the people they are designing for, and how their needs and context might inform design implications. Next, the ideation phase uses these design implications to inspire and inform design concepts, after which the implementation phase involves prototyping and ultimately evaluating them [21, 24]. Central tenets of HCD include continuous iteration, as well as embracing ambiguity; that is, accepting that it is difficult to anticipate or control outcomes of the approach [9].

HCD practitioners rely on various methods (see [17] for overview) and strive to bring social science research rigor to their use, while recognizing that creativity, openness, and serendipity are essential for “improving some characteristics of the world” [14]. We used an HCD approach for this project because it was aligned with our goal to avoid replicating existing interventions—primarily designed for youths in high-resource settings—and to instead develop an intervention which accounts for Kenyan youths’ experiences.

### 2.2 HCD in ICTD

Countless ICTD researchers have adopted HCD’s commitment to placing people’s concerns at the forefront of the design process. Studies using the approach, primarily the understanding and ideation phases, are common [12, 43]. Toyama has even described human-centeredness as the “dominant approach” in the ICTD field [44]. Reviewing all studies that use the approach is outside the scope of this paper. Here, we draw attention to prior studies that used methods similar to those in our project: interviews, photo studies, and design workshops.

Interviews are a typical human-centered design method [17]. They have been used to guide the development of various interventions aimed at addressing a range of issues in multiple LMICs [12]. Representative studies include Sambasivan et al.’s efforts to understand the everyday lives of sex workers in Bangalore, India. Their findings informed the development of a “phone-based broadcasting system” which disseminates HIV prevention strategies [42]. Working in Pakistan, Batool et al interviewed imams, mosque secretaries, and government officials to learn about donations; they used their findings to design a prototype mobile application [5]. Okeke et al. interviewed health professionals in Kenya when developing a digital feedback system aimed at improving health communication [37]. An HCD approach guided Chidziwisano et al.’s design of *SmartChama*, a mobile application for Kenyan women who are members of informal groups that pool and invest money [11]. Similar to these studies, we interviewed stakeholders and used our findings to design an intervention. Unlike these studies, we did not design a mobile application. We also complimented our interviews with a photo study exercise.

This HCD method [17] is similar to photo-elicitation and photovoice; it involves giving people cameras, asking them to take pictures, and then talking about their photographs [18, 46]. These methods also allow participants to visually communicate their realities, and to have a more active role in research [18]. There are some prior examples of using visual methods in ICTD (e.g., [31, 49, 50]); however, the method is not as widely used in ICTD as it is in fields like public health. Bentley et al. have encouraged ICTD researchers to make greater use of visual methods, because they can provide researchers with a richer understanding of contexts they are designing for [7]. We agree with this recommendation, and detail how we use the photo study method in our project, particularly during the ideation phase.

Goals of this HCD phase include generating a range of creative ideas, which are based on findings from the understanding phase [24]. Design workshops are a co-design and participatory method frequently used for eliciting these ideas [19]. Workshops’ structures vary: most require participants to engage in exploratory and generative activities that result in new design opportunities, often in the form of sketches or low-fidelity prototypes. Relevant examples include Hudson et al.’s co-design of tools to support speech and language therapists in Ghana; workshop outcomes included concept for mobile applications that support information sharing among speech-language pathologists students [22]. At the 2018 AfriCHI conference in Windhoek, Namibia, Wojciechowska et al. led a design workshop to incorporate African perspectives into concepts for drone use. The workshop produced prototypes of a “nurse drone” that reminded people to take medications [48]. In Kibera, Kenya, Barbareschi et al. held a design workshop to explore the future technologies desired by Kenyans with visual impairments, including a digital walking cane [4]. Within ICTD, less attention has been devoted to using HCD to redesign non-digital technologies. Given the field’s focus on computing, this is unsurprising. However, by focusing on non-digital technologies—in our project, a diary—we draw attention to alternative design outcomes of the HCD process.

#### 2.2.1 HCD, mHealth and Diabetes.

Enthusiasm for using HCD continues, and its methods are increasingly used in other fields, notably global health [8]. Latif et al. argue that mobile devices offer “unprecedented opportunity to transform the health services available to people across the globe”; they also suggest HCD as an effective strategy for ensuring these interventions align with LMIC cultural norms [27]. Holeman and Kane thoughtfully argue that HCD can improve global health equity “in a digital age”; funders such as USAID and the Bill & Melinda Gates Foundation have endorsed HCD and provided significant financial support for projects using the approach [21].

Similar to the aforementioned ICTD projects, these studies primarily use HCD to develop digital interventions, including mobile phone apps [6]. The growing enthusiasm for using computing to improve health outcomes, likely contributes to this design focus. However, as previously mentioned, a tolerance for ambiguity is a central tenet of HCD. In this paper we demonstrate how embracing ambiguity allowed for a non-technical intervention to emerge.

Finally, HCD has been described as a “promising approach for diabetes prevention” [29]. It has been used to design diabetes awareness programs with American adolescents [41], redesign insulin pens [47], and develop an “integrated model of group care” for Kenyans with diabetes and other non-communicable diseases [28]. These studies call for further research integrating the approach. Here, we implemented the understanding and ideation phases of the HCD process to develop a prototype intervention for Kenyan youths with T1D.

## 3 OUR STUDY

This paper details research conducted between April 2022 and September 2022. This project was a collaboration between two professors, their longtime Kenyan collaborator, designers, and healthcare workers affiliated with the Kenya Diabetes Management & Information Centre (DMI). a Nairobi-based NGO that works to raise awareness about T1D. The project was supported by a grant from the National Institutes of Health (NIH), specifically through a program that funds research to “to study the development (. . .) of innovative mobile health (mHealth) interventions (. . .) suited for LMICs.”^1^ In line with this program’s goals, we originally planned to use HCD to develop an mHealth application for Kenyan youths with T1D. However, as we detail here, a mobile intervention did not respond to participants’ context and needs. In other words, this intervention would *not* have been a human-centered outcome. Instead, we describe the process which resulted in these outcomes: community-building and a paper-based diary.

### 3.1 Study Setting

Recent reports suggest that 18.7 million people in Africa will be affected by diabetes by 2025 [35]. Similar to other African countries, T1D diagnoses are rising in Kenya, particularly among middle-to-late adolescents (13–18 y.o.). An estimated 50,000 to 70,000 Kenyan youths have T1D; however, the actual number is likely higher [40]. These factors, along with the authors’ familiarity with the country, were the primary reasons we chose Kenya as our field site.

We collected data at two distinct locations: Vihiga County, a lower-income agricultural region in western Kenya, and Nairobi, the country’s capital and largest city. Rural areas like Vihiga County typically have fewer job opportunities, lower population densities, and limited infrastructure (e.g., limited to no access to electricity and the internet). In contrast, urban areas like Nairobi generally offer better access to healthcare, and have more reliable electricity and internet. Collecting data at both a rural and an urban site provided insights into how we might design an intervention for youths across the country.

### 3.2 Participants

The Kenya Diabetes Management & Information Centre (DMI) maintains a database with approximately 400 youths with T1D. To ensure diverse perspectives, we recruited equal numbers of girls and boys, as well as participants from different socio-economic backgrounds (economic class, religion, and ethnic group). DMI identified 23 youths (between 11 and 18 y.o.), who had been living with a T1D diagnosis for at least one year, lived in one of our field sites, and were able to participate in every phase of the project. 12 youths were from Nairobi (8 boys, 4 girls) 11 youths were from Vihiga (6 boys and 5 girls). We invited participants’ caregivers to participate, because they tend to play a significant role in youths’ management of the condition (n=25). Similar to the youths, roughly half of these participants lived in Nairobi (n=13); however, most of these individuals were women (n=23). These participants participated in both project phases. Engaging with stakeholders (that is, individuals who have a direct interest in a project’s outcomes) is a key principle of HCD [24]. Doctors, health workers, and schoolteachers (n=10) were interviewed during the project’s understanding phase, because they frequently interacted with youths

### 3.3 Positionality and Ethics Approvals

Here we share aspects of our cross-cultural and interdisciplinary team’s positionality. The first and second authors are trained as a designer and geographer respectively, and have over 50 years of combined experience living and conducting research in Africa, including HCD projects in Kenya [51, 52, 55]. They collaborated with the third author, a Kenyan educated in the US, with extensive experience conducting interviews, organizing, and facilitating design workshops. The fourth author has lived with T1D for over 20 years, and is a diabetes educator and respected expert on the condition. Our team also included three Kenyan-based designers, with expertise in user research and graphic design. We collaboratively developed the interview protocol and workshop agendas, recruited participants, conducted interviews, analyzed data, and discussed design decisions.

The project received ethics approvals from the African Medical and Research Foundation (AMREF), the Kenya National Commission for Science, Technology and Innovation (NACOSTI), and Michigan State University’s institutional review board (IRB). Researchers provided and read aloud consent forms to participants, before conducting interviews and asking them to participate in the design workshops. These forms included language indicating that participants would share the photographs and sketches in this paper. Financial incentives were offered, including the digital camera used in the photo study exercise (valued at $35 USD) as well as 3,000 Kenyan shillings (about $22 USD) for participating in the ideation workshop. Transportation was provided to and from the local hotels where the workshops were held; meals and snacks were also provided.

## 4 Human-Centered Design: Understandings, Ideation and Prototype Development

As previously mentioned, the HCD process generally includes the understanding and ideation phases. Here, we describe the design and analysis methods used during these phases, as well as those used to develop our prototype.

### 4.1 Understand

In April 2022 our research team conducted semi-structured interviews with youths (n=23), and their caregivers (n=25) at their homes in Vihiga County and Nairobi. We also interviewed 10 people who frequently interacted with youths with T1D (i.e., doctors, community healthcare workers, and schoolteachers). These interviews were conducted separately; however, we used a similar interview protocol for each. We began sessions by collecting demographic data, and then asked youths and caregivers questions about their T1D diagnosis and about how they managed the condition. Given our initial focus on developing an mHealth application, all interviews included questions about phone use and accessing information via the mobile internet. During this phase, we also used the photo study method [17] to collect participants’ visual and self-reported insights. Following the interviews, we gave each youth a digital camera and instructions on how to use it. We encouraged participants to take pictures of their daily activities related to diabetes.

### 4.2 Ideation

In June 2022, we returned to both sites and held day-and-a half-long design workshops with the same youths and caregivers from the understanding phase (24 participants in Vihiga; 23 in Nairobi). To accommodate participants’ schedules, these sessions were held during school breaks and took place at local hotels. We used focus group discussion, brainstorming, and sketching methods to learn more about youths and T1D management and to generate design ideas. During workshops we also gave youths printed copies of the photographs from the photo study and asked them to choose a subset to discuss.

Parents and youths were divided into separate groups; there were six groups in both workshops (12 groups total). The groups were encouraged to discuss themes from youths’ photo presentations (reported in the findings), think about how the themes might translate into ideas, and describe which challenge(s) the idea would address. Next, the groups were asked to choose their favorite idea and draw it. The workshops concluded with groups presenting their final ideas; presentations lasted between 5 and 15 minutes and were followed by a brief discussion period. Mary Karanu facilitated the workshops and recorded insights from each groups’ presentations on an easel pad with sticky paper. We also video recorded these presentations and took field notes.

### 4.3 Prototype Development

The researchers, project collaborators and designers participated in de-briefing sessions immediately after both workshops. Based on their observations during the workshops (see findings in Section 5.2), the team collectively decided to develop a diary. The designers drew from the data collected during the workshops, especially their observations, and their conversations with youths, their caregivers, and the medical doctors, to rapidly develop three initial versions of the diary. Next, we engaged in parallel prototyping: that is, the process of simultaneously considering these three ideas before refining one [13, 17]. Afterwards, the authors and designers engaged in bi-weekly meetings (via Zoom) and countless conversations (via WhatsApp) to iterate upon and refine the diary shown in Figure 3.

### 4.4 Analysis

Data analysis occurred throughout our project; a similar approach was used to analyze data from the understanding and ideation phases. The data included the authors’ field notes, interview transcripts, photographs, sketches, themes recorded on sticky notes, audio recordings, and videos. Interviews and groups presentations were digitally recorded, and transcribed verbatim (and, when necessary, translated into English) by experienced Kenyan transcribers. Goals of our analysis were consistent with HCD methodology; we analyzed data to develop an understanding of youths’ and their caregivers’ experience living with T1D, in order to design an intervention that responded to their needs and context [24]. Manual content analysis was used to identify themes [17].

We also used this approach to analyze participants’ photos and sketches. We reviewed the 854 photos taken with the digital cameras, and the 12 sketches and pages of notes from the workshops, and looked for consistent themes. Insights from the photo and sketch analyses were integrated with interview data to develop the findings presented here. The authors consulted with each other (in person, and via WhatsApp and Zoom) to clarify and confirm themes in the data and to design the prototype diary. A more detailed description of our research design and data analysis procedures can be found here [57].

## 5 FINDINGS

Here, we present findings from our understanding phase; in particular, participants’ T1D-related challenges and limited smartphone use—an observation that led us to avoid designing an mHealth app. We then present participants’ design concepts from the ideation phase. Next, we describe how these findings informed the development of an early prototype diary. Throughout this section, we also note observations about participants’ experiences in the project.

### 5.1 Understanding

Analysis of interviews with youths, their caregivers, and key stakeholders, as well as insights gleaned from participants’ photos, provided us with an understanding of the context we were designing for. We found that youths and their caregivers knew little about T1D prior to diagnoses, stigma and misunderstanding about the condition were common, and economic and infrastructural challenges hindered youths’ consistent access to insulin and proper food. We also learned about the challenges associated with owning and using smartphones.

All youths had been living with T1D for at least one year, and more than half had been managing their condition for four or more years, including one 17-year-old boy who was diagnosed at age six. Prior to the diagnosis with “sukari” (Swahili for sugar), both the youths and their caregivers knew little about diabetes. When recounting their initial diagnosis, all participants clearly remembered what led up to it, typically describing frequent urination, extreme thirst, and fatigue—symptoms that prompted a visit to a doctor, where they were eventually diagnosed. Youths consistently told us they were “shocked” upon learning they had T1D, because the condition was commonly thought to affect older adults and people in higher-income countries. However, over time, and after learning more about diabetes, most participants accepted that, while managing the condition was demanding, they could lead a “normal life” if they ate properly, exercised, monitored their blood sugar levels, and regularly injected insulin.

**Figure 1:**
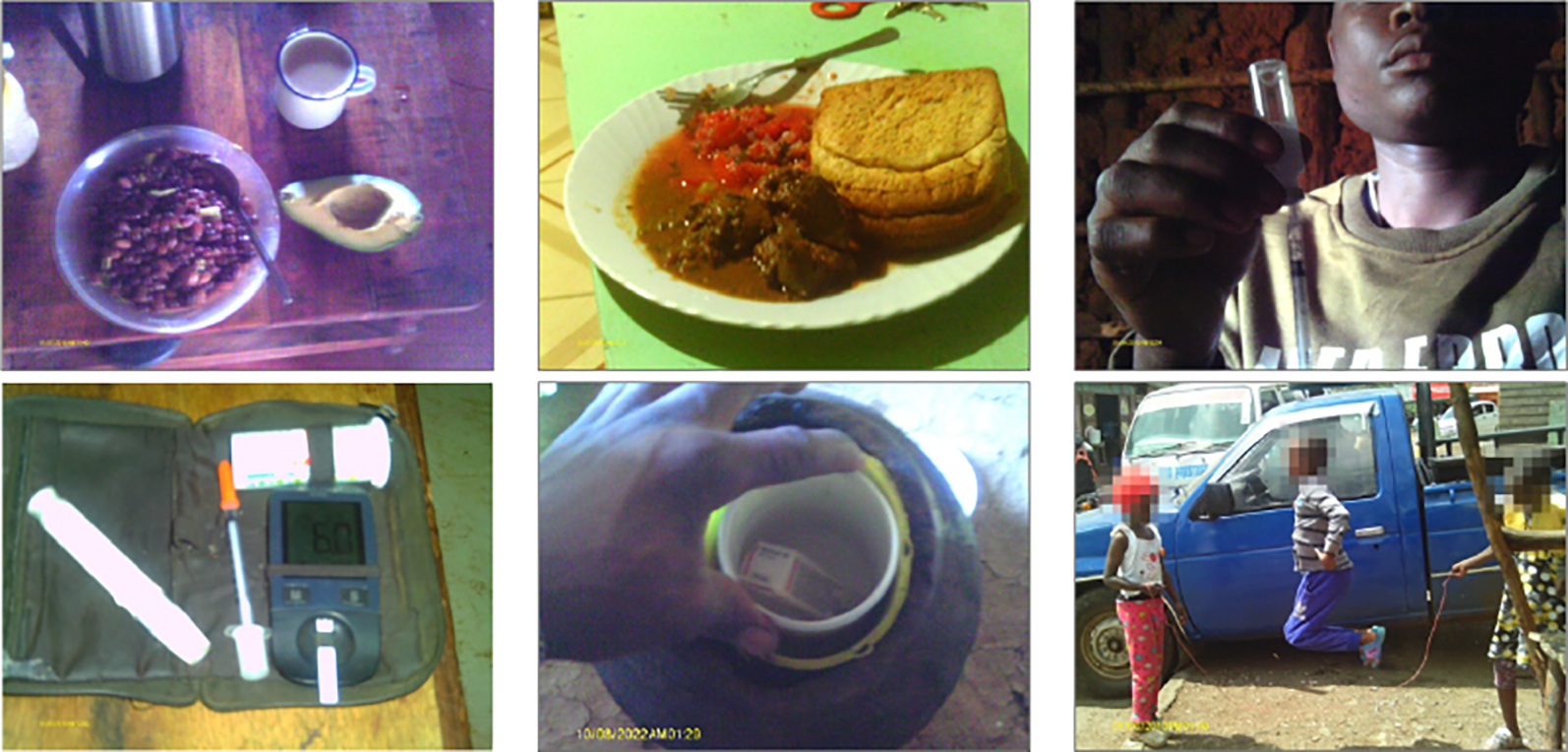
Selected Photos from Photo Study Exercise

Participants generally learned about T1D management through in-person interactions with DMI staff and healthcare providers; few relied on the internet to access T1D information. They understood how to manage their condition, including which foods to avoid, which to eat, and the importance of regularly checking their blood sugar levels, injecting insulin, exercising, and routinely seeing their healthcare provider. This knowledge was also captured in their photos. All participants completed the photo study exercise, though the number of photos taken varied—one youth took 192 pictures, while another took only three, with 37 being the average. The images frequently featured plates of recommended foods (spinach, cabbage, avocado, beans, brown ugali, etc.), bottles of insulin, and photos of youths playing football, working in their gardens, and other physical activities (Figure 1).

Although youths and their caregivers knew how to manage T1D, they consistently experienced challenges related to the condition. Participants frequently described feelings of loneliness and “not fitting in,” because they did not know others with T1D. Negative stereotypes and misunderstandings about T1D, especially in school where youths spent a significant amount of time, contributed to this sense of isolation. Approximately a quarter of participants did not share their diagnosis with classmates to avoid feeling further marginalized. Those who did share this information reported being avoided by others, who believed T1D was contagious (it is not). Some youths even faced accusations of injecting illegal drugs when they were actually administering insulin. Teachers generally confirmed these findings and said that there was a need for more education about T1D in schools. Youths also said that they responded to these misconceptions by educating others about diabetes.

There were other challenges. Insulin access was sometimes problematic due to intermittent pharmacy supplies. Doctors told us that administering insulin injections three to four times a day provides the best way to control blood sugar levels, but most youths in our study only inject insulin twice a day. It was also common for them to skip doses when they did not have the medication. Storing insulin presented another challenge. The drug is temperature-sensitive and loses potency when not kept cool or when exposed to sunlight. Kenya’s hot climate, combined with some participants’ limited access to electricity—and therefore home refrigerators—made it difficult to store insulin at the appropriate temperature. Participants’ photos documented the clay pots and other improvised cooling systems they used to store insulin (Fig. 1, bottom row, middle).

Caregivers’ responses to our questions drew attention to systemic problems that made T1D more challenging, particularly the cost of treatment. Inconsistent employment caused parents to worry about the financial costs associated with T1D, including the expense of the recommended diet, travel to clinics, and medication. Some caregivers, especially those from lower-income households, described making trade-offs to afford medication, such as forgoing essential items and selling assets [32]. The word “stress” frequently appeared in interview transcripts, as illustrated by this father’s statement:

If I can get a place to work and get money to sustain us, but you can’t do all of this without money, it is money—money is everything. It just causes stress. Money to get food, she had been told eat a lot of vegetables and eat fruits, but they are expensive, vegetables are expensive.

These findings draw attention to the confluence of factors—social, infrastructural, and economic—that affected participants’ T1D experiences.

#### 5.1.1 Redefining the problem:

Limited Smartphone Access. Other findings prompted us to redefine our project’s focus, as is typical in HCD [24]. As previously mentioned, the project was funded by an NIH program aimed at developing “innovative mobile health (mHealth) interventions.” However, a digital intervention did not support participants’ needs or context, primarily due to limited smartphone ownership and internet access. Mary^2^, a mother of a 17-year boy with T1D, captured these challenges; we paraphrase her response to a question about the mobile internet and smartphones:

So many people don’t have Wi-Fi or bundles^3^, so if we want to reach the people in rural areas (. . .) there are people who don’t have internet, bundles, for them it is an unnecessary luxury so that is one challenge (. . .) Of course for an app you will need a smartphone, you will need a couple thousands of shillings to purchase one—the cost is an issue (. . .) And then accessibility in schools. Our issue is the youth like my son is in boarding school. How will he use in school if it is banned?

Her response summarized challenges discussed by others. 35 of the 48 participants had mobile phones; however, there were significant disparities between rural and urban participants’ access to these devices. 10 of the 12 Nairobi youths reported having a mobile device; seven reported owning smartphones. Their caregivers also had mobile phones; all 13 reported owning a device, and 10 had smartphones. In Kenya, a low-quality smartphone can be purchased for 2,500 to 3,500 KES (about $20-$27), yet these devices remain prohibitively expensive, especially for participants living in rural areas. In Vihiga County, only 4 caregivers and 1 youth reported owning a smartphone. Instead, it was more common for these participants to own inexpensive basic or feature phones, popularly known as “kabambe.”

mHealth efforts are dependent on owning a smartphone and being able to consistently use it to access the internet [26]. Using these services also requires owning a smartphone and having sufficient data bundles—both of which were lacking, particularly among participants in rural areas who were more likely to own basic or feature phones. These challenges have been repeatedly documented in prior studies of mobile phone use in Kenya [51, 53, 56]. Additionally, youths with phones could not regularly use them because mobile devices are banned in Kenyan schools, a common practice in African countries [58]. Teachers explained that these bans are in place to prevent distractions and address inequitable access to mobile phones. As a result, developing a mobile phone application would not have addressed the participants’ needs or context in a human-centered way.

### 5.2 Ideation

Here we describe how our research team, study participants, and designers decided to develop a paper-based diary by presenting outcomes from two design workshops. We begin with findings from the 23 youths’ descriptions of their photos, then focus on the design ideas created by groups of caregivers and youths, along with observations on how the workshops supported community building.

Both workshops followed the same protocol, beginning around 9AM. After a prayer, introductions, a review of the project’s goals, updates on its progress, and obtaining informed consent, participants engaged in an icebreaker activity. The introductions were important because, although members of the research team had met participants individually, the participants had not met each other. Icebreaker activities, such as forming the word “coconut” with their bodies, elicited laughter and helped establish the workshops as spaces where participants felt comfortable sharing ideas during the subsequent activities.

A significant part of the workshops was devoted to participants explaining their photos [17]. The youths discussed pictures of themselves using glucometers, injecting insulin, eating food, and exercising, emphasizing how these activities were crucial to their well-being. They also shared selfies and reflected on the challenging aspects of T1D, such as the pain of injections, as well as positive moments, including celebrating successful blood sugar management or simply managing their condition over the years. In one participant’s words:

I love this photo because it tells me how long I have been living with the disease. It gives me hope and courage to continue living with it.

Participants in both workshops reiterated findings from the understanding phase by sharing stories about the challenges of keeping insulin cool, accessing healthy foods, and experiencing stigma in school. These anecdotes seemed to help participants learn more about their shared experiences and realize they were not alone in managing T1D, as evidenced by the sense of camaraderie that emerged during the workshops. Participants frequently nodded in agreement, as they discussed their challenges and coping strategies throughout the workshop.

The photo presentations also stimulated discussions among groups as they developed design ideas. We observed groups engaging in deep conversations, writing down their shared thoughts, exploring how to translate them into artifacts, and then working to visualize them. Prior research consistently suggests that visual research methods can improve a community’s understanding of their needs, foster trusting relationships, and elicit open and honest conversations, especially among marginalized groups [10]. This seemed to occur during the workshops, as one youth expressed:

I have more self-confidence, as I have met other children who are suffering like me. We have been able to make friends.

These findings draw attention to how the HCD process facilitated not only creative problem-solving but also a sense of belonging and mutual support among participants.

#### 5.2.1 Design Concepts.

**Figure 2:**
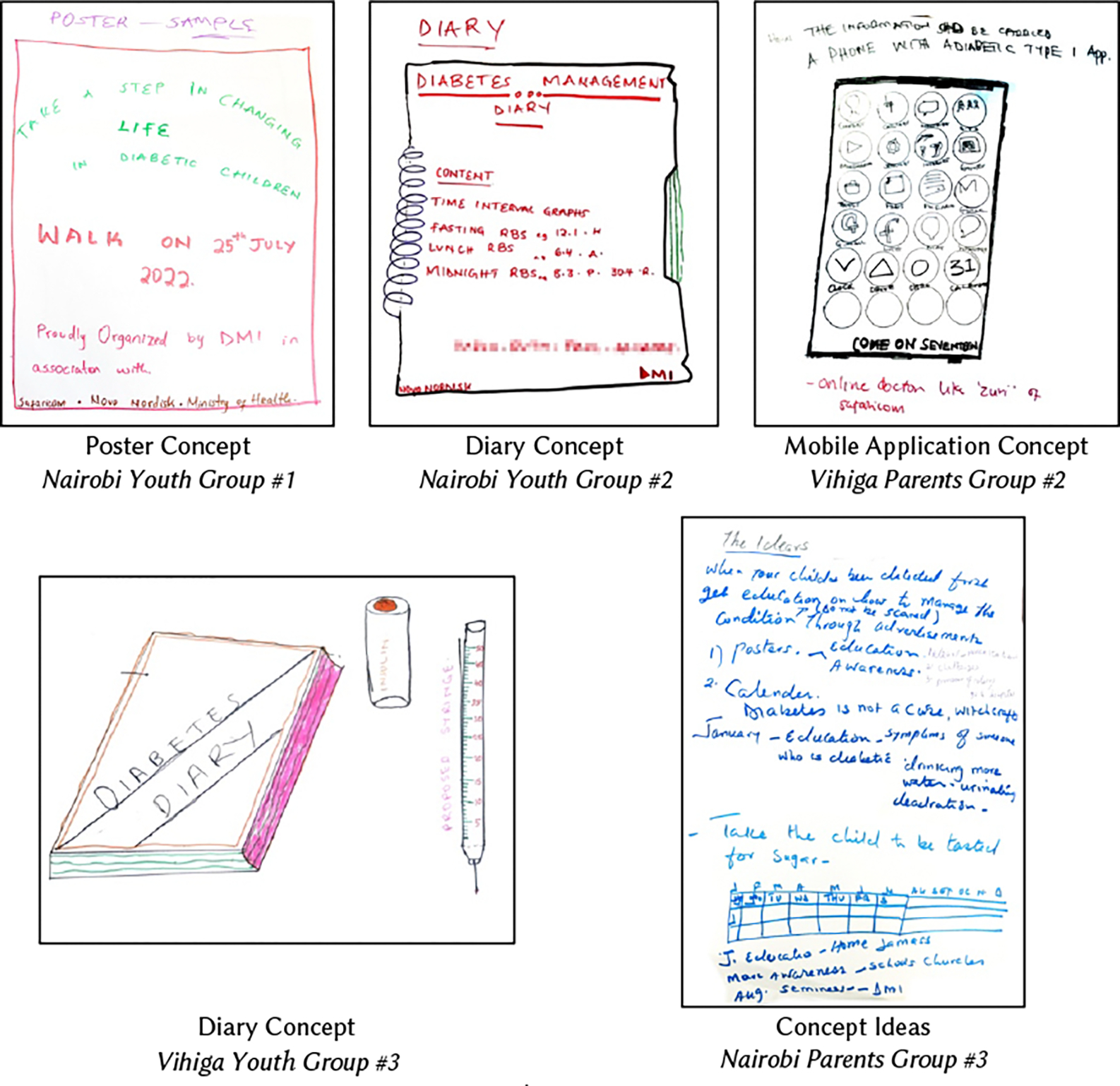
Participants’ Design Sketches and Ideas

Towards the end of the second day of the workshops, groups presented their ideas. Although we encouraged groups to develop and draw concepts—foundational ideas that guide the development of a design—some groups were more successful at this than others. Broadly, the youths tended to draw concepts, like those shown in Figure 2, while parents tended to write out ideas (Figure 2, bottom right). All 12 groups presented at least one idea and most presented two to four. One Nairobi youths group listed seven concepts, including a calendar, poster, comic book, and youth camp. Other groups, particularly the parents, tended to elaborate on challenges learned during the understanding phase, such as the need for greater awareness about T1D in schools and more support for securing insulin and accessing healthcare.

Strictly speaking, half of the teams presented artefacts that already existed or that they needed (e.g., glucometers, new shoes, and refrigerators). Other groups shared more novel ideas during the presentations, such as a “piki-piki” (motorcycle taxi) service that delivers insulin to youths, and using “Zuri,” a virtual artificial intelligence (AI) chatbot [25], to seek medical advice. Rather than generating design concepts, it was more common for groups, particularly caregivers, to discuss the need for government interventions to improve access to medication, healthcare, and livelihood opportunities. These quotes are from their presentations:

We are saying that the government should provide this medication for free.

Our main issue is storage, so my idea, my proposal was to let the ministry find ways to help us store our insulin by providing ice boxes or these traditional freezers.

So, the government, the ministry, might come up with some income-generating programs for people, so that will be able to buy food and other resources they need to manage their type 1 diabetes.

HCD involves navigating constraints, particularly those related to time and resources [14]. Within our project’s year-long period and budget, and considering the funding agency’s expectation that an artifact would be designed, we recognized that while some of these suggestions might lead to interventions that genuinely addressed participants’ needs, they were not feasible project outcomes. Five groups (three in Vihiga and two in Nairobi) described and/or sketched a diary (Figure 2), a concept that we could develop within the projects’ constraints.

When discussing this concept, groups consistently described how a diary could address multiple needs: reminding them of doctors’ appointments, documenting the foods they ate, and tracking their blood sugar levels. Many youths also believed that a diary could educate others (e.g., schoolteachers) about T1D. Healthcare workers also found this concept promising, because patients could record information they could use to adjust treatment plans. These stakeholders also hoped that youths who were encouraged to use a diary to track their daily habits and moods, could more effectively identify patterns and trends that may affect their blood sugar. For example, youths might notice that eating certain foods consistently causes high blood sugar, or that worrying makes their blood sugar levels drop.

During discussions about the diary, the significance of including a “Time-in-Range” (TIR) graph emerged. TIR is an easy-to-understand measure that shows how much time (e.g., hours per day) a person’s blood sugar levels stay within a healthy “target” range (usually between 4 and 10 mmol/l). Most people with T1D should aim for a TIR of at least 70% of readings, meaning they should strive to be in range (neither high nor low) for roughly 17 out of 24 hours each day [1]. Adolescents in high-income countries tend to rely on continuous glucose monitors to calculate and visualize TIR; however, youths in our study would need to manually calculate and chart their levels using graph paper, as none had access to these digital devices. Eric Omondi, a diabetes educator and member of our research team, was especially enthusiastic about including this feature in the diary because manually tracking TIR helped him become more aware of his own blood glucose levels.

Group discussions about this concept guided other design decisions, including the choice to adopt a comic book aesthetic similar to the one used in *Shujaaz* (a popular Kenyan comic book that includes stories about how youths navigate challenging life and health issues) [23]. Youths in both groups talked about the benefits of journaling; that is, writing about their weekly experiences managing their condition. Workshops participants also decided the diary would need enough pages for them to document their blood sugar levels for three months; that is, the time between their doctors’ appointments.

#### 5.2.2 Participants’ Workshop Experiences.

As previously mentioned, participants appeared highly engaged in the workshops and expressed tremendous gratitude at the conclusion of these events. This gratitude was directed toward the doctors, the research team, and God. Youths and their caregivers consistently told the research team that they appreciated the opportunity to learn more about T1D, to contribute to the project, to eat and stay at the hotels, and most significantly, to meet each other. The workshops concluded with participants exchanging phone numbers and planning to meet again. One participant expressed their gratitude in a song; we include excerpts here:

This disease diabetes has made us worry. When we thought it only affects, only adults; boundaries it lacks, affecting anyone, poor and rich, all in the same boat.

We say thank you very much; to the school of [blind for review]; you’ve made us very new, until we’re confident; good places we’ve arrived, for the dedication you have. We’ve drunk, we’ve eaten, just like the honorable.

Because of you all, we expect to go further, in spite of type-1 diabetes. We will follow through guidance To enable us to go further. Good diet and the others. All are in our minds.

The gratitude and connections among participants highlight how HCD methods, particularly workshops, can support community-building and other outcomes that are not necessarily design-related.

### 5.3 Prototype Development

As previously mentioned, there was strong agreement among the researchers and designers that the diary was the most promising concept from the workshops. The concept addressed multiple needs identified during the understanding phase and reiterated during the design workshops, particularly those related to limited access to smartphones and the need to educate the broader public about T1D. The designers envisioned creating a diary that would allow youths to carefully document their blood sugar levels, foods eaten, and other relevant information. They looked at other examples of diaries and journals developed for people with T1D to inform their design process. Additionally, they considered incorporating a poster into the diary to help educate others about T1D.

**Figure 3:**
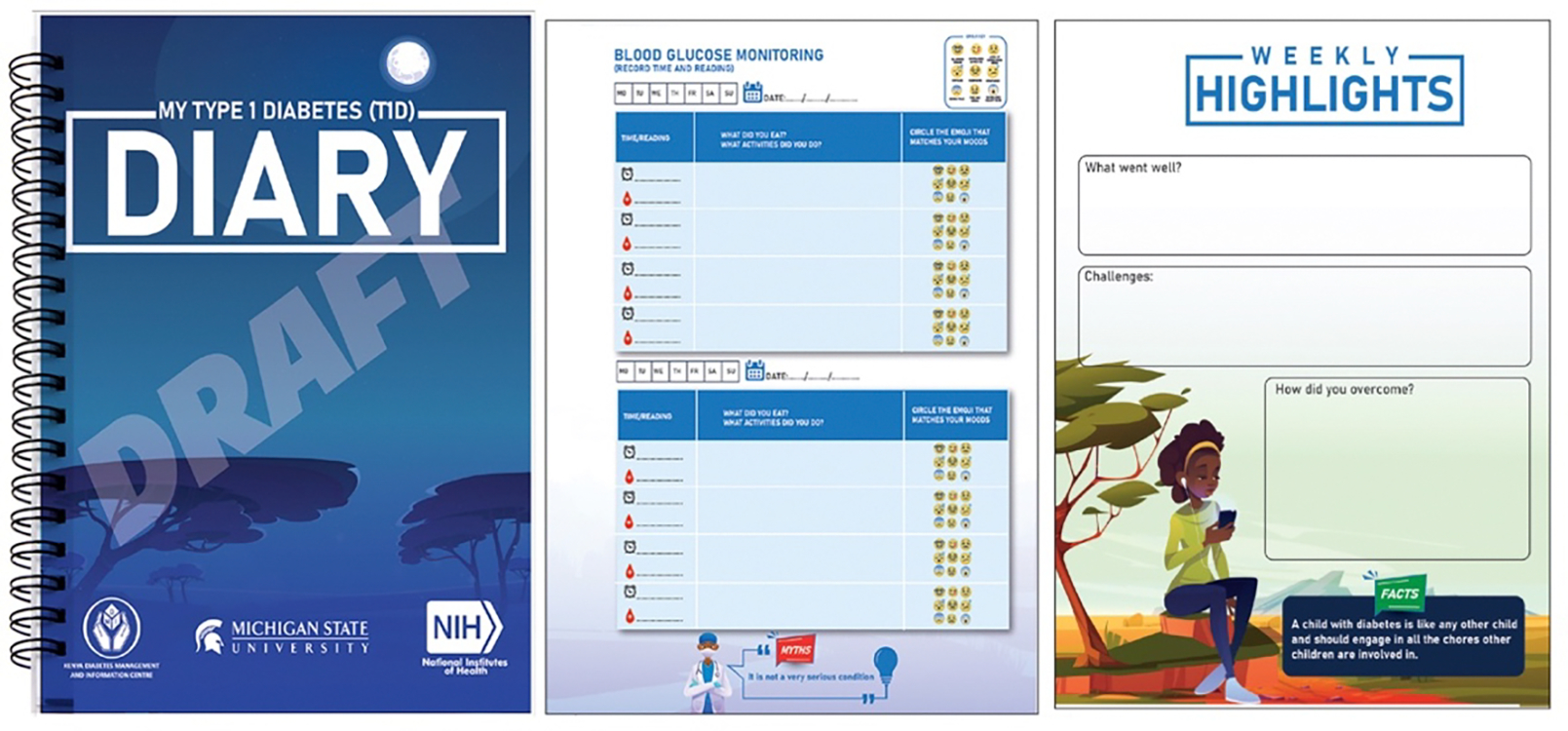
Diary Prototype with Cover and Selected Pages (i.e., Blood Glucose Monitoring and Journaling)

**Figure 4:**
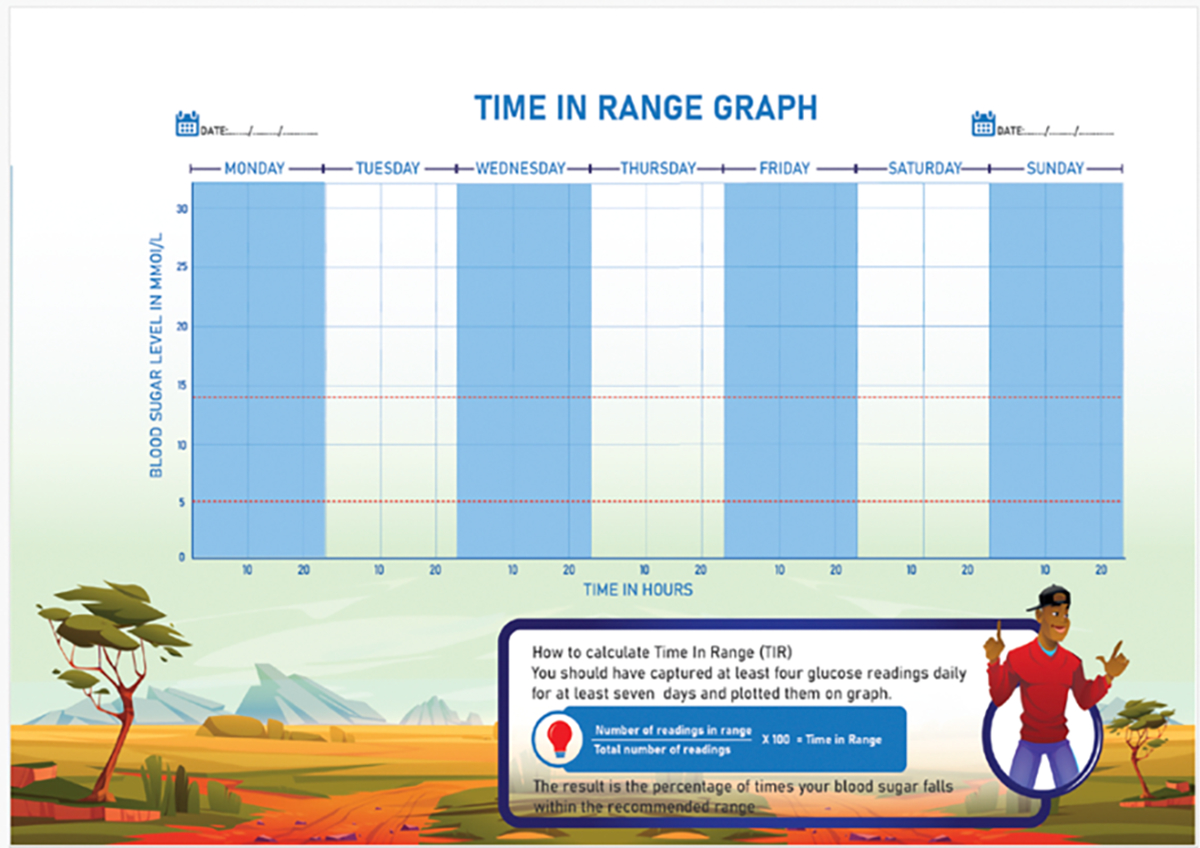
Time In Range (TIR) page from Diary

**Figure 5:**
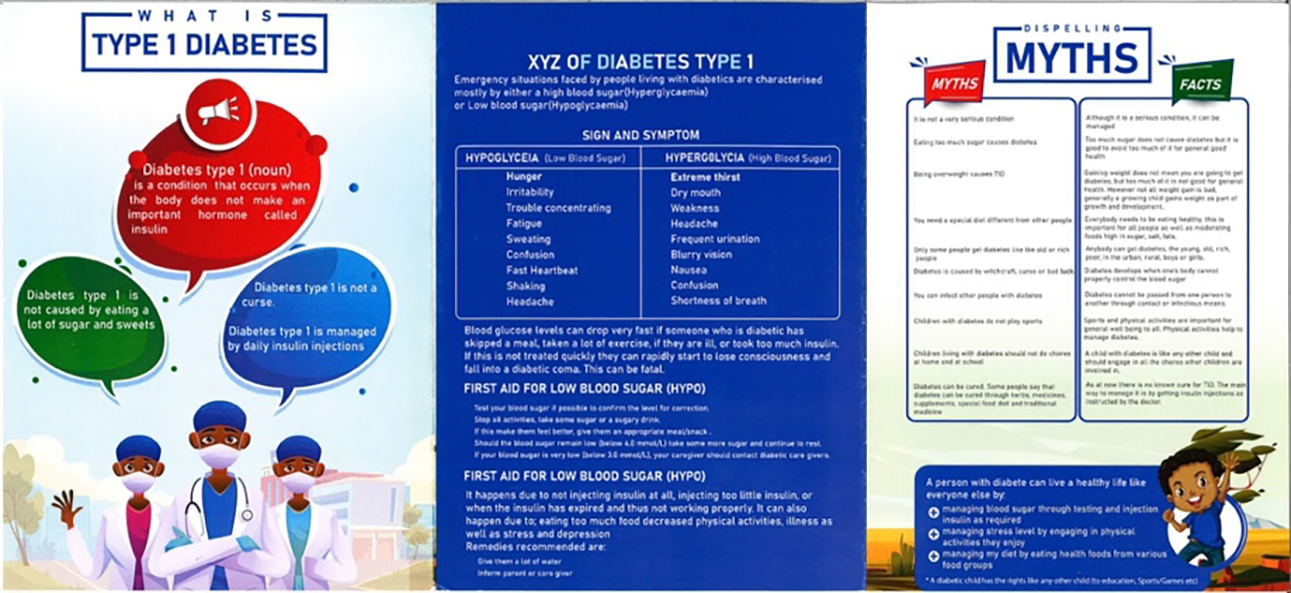
Educational Poster Insert from Diary

The designers incorporated key features discussed during the workshops into their initial concepts (e.g., TIR, blood sugar monitoring, and journaling pages) (Figures 3 and 4). They also used their personal judgment to make decisions about the diary’s cover and illustrations. One designer added emojis to the blood sugar monitoring page, believing this feature would appeal to youths. Youths could circle an emoji that corresponded with their mood when recording their blood sugar levels. The designers were also mindful of the need to educate others about T1D, and included dedicated pages in the diary that provided information about the condition, as well as a poster insert that could be displayed at schools (Figure 5). This prototype was completed in September 2022 (Figure 3).

## 6 DISCUSSION

HCD is widely used in ICTD projects to develop digital technology interventions for people in low- and middle-income countries (LMICs). We applied HCD to collaborate with Kenyan youths, their caregivers, and a local design team to develop a prototype paper-based diary to support youths in managing their T1D. This paper contributes a novel case study detailing our project’s understanding and ideation phases, as well as the development of our initial prototype. Outcomes from our design workshops further demonstrate that members of marginalized communities are experts on the interventions they need and should be treated as such [19]. We also provide additional support for the use of visual methods (i.e., photo study) in ICTD.

Significantly, we learned that developing a digital intervention (i.e., an mHealth app) would *not* be a human-centered outcome, because it was not aligned with participants’ needs or context. Many participants did not own smartphones, and those who did rarely used them to access the mobile internet. Analog interventions are uncommon outcomes in HCD within ICTD. Here, we emphasize the importance of considering these and other non-digital outcomes when using HCD in ICTD, such as new knowledge creation and community-building. These outcomes are crucial because they are immediate, whereas—in the case of this project—further research is needed to evaluate the diary’s impact on youths’ T1D management. Finally, we discuss the limitations of our project in this section.

### 6.1 Expanding HCD to Consider Non-Digital Outcomes

The vast majority of HCD projects result in recommendations for and prototypes of digital technologies [5, 42], including prior human-centered studies conducted in Kenya [11, 37]. The emphasis on digital, especially mobile technologies, and the focus of our project’s funders, appears to be driven by the widespread belief in their revolutionary potential to improve health and other conditions in LMICs [21, 27]. However, the evidence supporting the transformative effects of these devices in improving health outcomes is mixed and limited [6, 36].

We suggest that a more authentic HCD process—one that truly embraces ambiguity and responds to users’ needs and contexts, including the well-documented challenges associated with mobile phones [51, 53, 54]—should include attention to non-digital interventions. Less focus on digital outcomes might create opportunities to explore other non-digital interventions, such as the government initiatives mentioned by our participants or new forms of insulin that do not require refrigeration. Funders supporting HCD efforts should also reconsider starting with a specific technology or solution already in mind and recognize that, strictly speaking, this is counter to a human-centered approach. In other words, digital intervention should not be assumed as the default outcome of the HCD process.

### 6.2 Finding Value in the Process: New Knowledge and Community Building

T1D diagnoses are rising in Kenya, and elsewhere in Africa; however, there is a paucity of research—especially qualitative studies and those focusing on adolescents—on the condition [34, 39]. Findings from our understanding phase generally aligned with those reported in prior studies of T1D management in LMICs (e.g., [30, 32, 33, 38]). Our use of HCD methods, particularly photo study and design workshops provide more nuanced understanding of these issues that interviews alone rarely capture. ICTD scholars have previously described HCD as a distinctive inquiry practice [52]; that is, a unique method of investigation that is set apart from others. Our research further supports these observations.

In addition to providing researchers with a deep understanding of the underserved communities’ lived experiences, HCD methods appeared to support community-building among participants. The project, especially the workshops, provided a way for participants to connect with others facing similar struggles and appeared to reduce feelings of isolation and stigma that many experienced. This was an immediate outcome of our project, and one that we speculate might be longer-lasting and more impactful than the diary itself. More efforts are needed to explore how to measure this impact as an outcome of the HCD process.

### 6.3 Project Limitations and Future Research

Of course, the long-term outcomes of our process are unknown, and research to evaluate the diary is needed. HCD projects have been critiqued for providing limited—or no—rigorous evaluation of project outcomes [6, 20]. This project may be yet another example of an HCD initiative that is never fully implemented or adopted; future research is required to assess the diary’s impact. While our study focused on T1D in Kenya, its implications may extend to other LMICs. This merits further investigation. More broadly, the project’s fast-paced nature and focus on developing an intervention meant that some systemic problems related to T1D were overlooked (e.g., the need for places to store insulin). We urge ICTD researchers to balance the benefits of HCD with these limitations.

## 7 CONCLUSION

In this article, we document the initial phases of our project’s HCD cycle—from the formative research phase, through ideation, to developing an early prototype of a paper diary aimed at supporting Kenyan youths in managing T1D. We provide a reflective account detailing our methodological choices and findings, along with a discussion of the potential benefits and drawbacks of using HCD in LMICs. Our conclusion is that HCD is a valuable approach that can lead to novel interventions to improve Kenyan youths’ ability to manage their T1D. However, we also conclude that design outcomes are not the most significant benefit of HCD; rather, the process is valuable for highlighting the limitations of digital technologies and fostering community among marginalized groups.

## CCS Concepts

• Human-Centered Computing; • Human Computer interaction (HCI); • Empirical studies in HCI;

## Footnotes

1 Detailed information about this program can be found here: https://www.fic.nih.gov/Programs/Pages/mhealth.aspx

2 All participants’ names have been replaced with pseudonyms to preserve their anonymity.

3 In Kenya, “data bundles” or airtime are prepaid, or postpaid packages offered by mobile network operators that provide users with a specified amount of data for accessing the internet on their mobile devices.
